# Interferons reshape the 3D conformation and accessibility of macrophage chromatin

**DOI:** 10.1016/j.isci.2022.103840

**Published:** 2022-02-01

**Authors:** Ekaterini Platanitis, Stephan Gruener, Aarathy Ravi Sundar Jose Geetha, Laura Boccuni, Alexander Vogt, Maria Novatchkova, Andreas Sommer, Iros Barozzi, Mathias Müller, Thomas Decker

**Affiliations:** 1 Max Perutz Labs, University of Vienna, Vienna, Austria; 2 Vienna Biocenter Core Facilities GmbH (VBCF), Vienna, Austria; 3 Institute of Molecular Biotechnology of the Austrian Academy of Sciences (IMBA), Vienna, Austria; 4 Research Institute of Molecular Pathology (IMP), Vienna Biocenter (VBC), Vienna, Austria; 5 Institute of Cancer Research, Department of Medicine I, Medical University of Vienna, Vienna, Austria; 6 Institute of Animal Breeding and Genetics, University of Veterinary Medicine Vienna, Vienna, Austria

**Keywords:** Molecular biology, Molecular mechanism of gene regulation, Epigenetics, Cell biology

## Abstract

Engagement of macrophages in innate immune responses is directed by type I and type II interferons (IFN-I and IFN-γ, respectively). IFN triggers drastic changes in cellular transcriptomes, executed by JAK-STAT signal transduction and the transcriptional control of interferon-stimulated genes (ISG) by STAT transcription factors. Here, we study the immediate-early nuclear response to IFN-I and IFN-γ in murine macrophages. We show that the mechanism of gene control by both cytokines includes a rapid increase of DNA accessibility and rearrangement of the 3D chromatin contacts particularly between open chromatin of ISG loci. IFN-stimulated gene factor 3 (ISGF3), the major transcriptional regulator of ISG, controlled homeostatic and, most notably, induced-state DNA accessibility at a subset of ISG. Increases in DNA accessibility correlated with the appearance of activating histone marks at surrounding nucleosomes. Collectively our data emphasize changes in the three-dimensional nuclear space and epigenome as an important facet of transcriptional control by the IFN-induced JAK-STAT pathway.

## Highlights

• Type I IFN and IFN-γ rapidly increase chromatin accessibility at many target loci
• Both IFN types cause rapid chromatin loop rearrangements at target loci
• Loop formation increases between regions of accessible promoter chromatin
• Large contribution of transcription factor ISGF3 to gene control by both IFN types

## Graphical abstract

**Figure:**
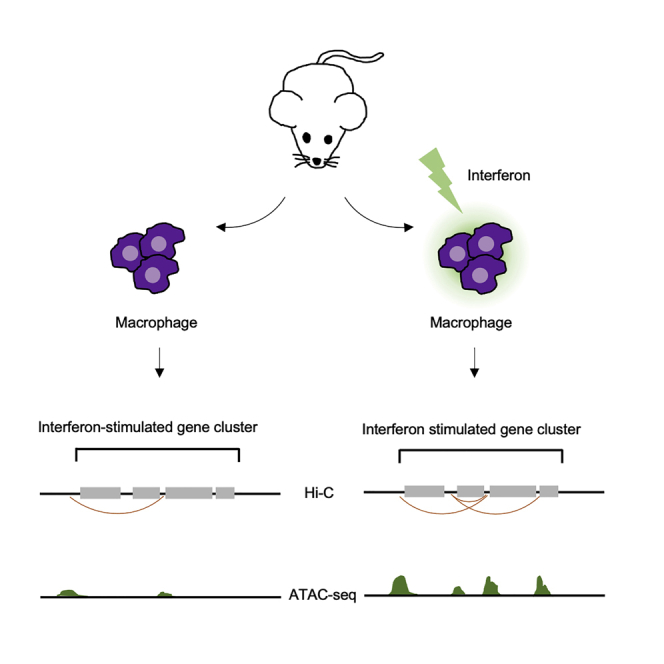

## Introduction

Interferons are soluble messengers that are produced in response to invading pathogens. Particularly the type I-IFN species IFN-α and IFN-β (collectively called IFN-I) are of vital importance in the course of cell-autonomous antiviral immunity. IFN-γ, the only member of the type II interferon family, is similarly capable of inducing the antiviral state, but functions predominantly as a macrophage-activating cytokine in the immune system (MacMicking, 2012; McNab et al., 2015; Schneider et al., 2014). Although IFN-I and IFN-γ have diverging functions in many biological scenarios, both activate the JAK-STAT pathway (Darnell et al., 1994; Levy and Darnell, 2002). The extent to which the two cytokines elicit different transcriptional profiles in cells is incompletely understood. Improved knowledge about nuclear responses to IFN-I and IFN-γ is required to decipher the relationship between transcriptome changes and the cytokines' immunological impact. Additionally, such insight will improve our understanding of the molecular hallmarks of interferon-associated diseases (Crow and Manel, 2015; Sancho-Shimizu et al., 2011).

The JAK-STAT pathway represents a striking example of receptor-mediated signal transduction (Stark and Darnell, 2012). This evolutionarily conserved pathway utilizes a simple membrane-to-nucleus mechanism for rapidly inducing gene expression (Au-Yeung et al., 2013). Thus, it serves as a simple paradigm for how cells sense extracellular signals and translate them to a specific transcriptional outcome. Signal transduction downstream of IFN-I receptors requires activated JAKs to cause the formation of the transcription factor IFN-stimulated gene factor 3 (ISGF3). This heterotrimer of STAT1, STAT2, and interferon regulatory factor 9 (IRF9), assembles at interferon-stimulated response elements (ISREs), present in the promoters of interferon-stimulated genes (ISG) (Ivashkiv and Donlin, 2014; Kessler et al., 1990; Schindler et al., 2007). The IFN-γ receptor on the other hand employs JAKs to activate the gamma interferon-activated factor (GAF), a STAT1 homodimer, which stimulates ISG expression by binding to gamma interferon-activated sites (GAS) (Decker et al., 1997).

Regulation of transcription in eukaryotic cells is based on a fine-tuned interplay between chromatin structure and recruitment of a plethora of transcription factors to enhancers and promoters (Maniatis et al., 1987; Smale and Kadonaga, 2003). These regulatory elements differ in their function and location with regard to the transcription start site (TSS), but share some common characteristics such as specific active epigenetic marks and their property to recruit the RNA polymerase to the core promoter and thus initiate transcription. However, putative enhancers cannot be predicted solely based on genomic proximity as gene expression programs are orchestrated within the three-dimensional nuclear space, thus often bridging considerable genomic distances (Bonev and Cavalli, 2016; Schoenfelder and Fraser, 2019).

Recently, genome-wide chromosome conformation capture (Hi-C) experiments have shown that chromosomes acquire their spatial order at several different levels of their structural organization (Dekker and Mirny, 2016). This three-dimensional folding of the eukaryotic genome is a highly organized process, tightly linked to transcription. At the megabase scale, chromosomes are organized into functionally distinct A and B compartments, representing transcriptionally active and inactive genomic regions, respectively (Lieberman-Aiden et al., 2009; Rowley and Corces, 2018). At the next level, chromosomes fold into topologically associating domains (TADs) and chromatin loops. Their formation depends on the ring-shaped, multiprotein cohesin complex that cooperates with the sequence-specific DNA binding protein CTCF (Buenrostro et al., 2015; Rao et al., 2017; Schwarzer et al., 2017; Wutz et al., 2017). TADs and loops contribute to the regulation of gene expression by facilitating the interactions between promoters and enhancers located within the same domain (Dixon et al., 2012; Dowen et al., 2014; Symmons et al., 2016).

Whether IFN induce changes in the genome organization in order to control gene expression is largely unexplored. Here, we investigate genome-wide chromatin dynamics in primary murine macrophages exposed to IFN-I and IFN-γ and find that they trigger a rapid rearrangement of the 3D chromatin organization on ISG-rich regions. Surprisingly, both interferon types cause highly overlapping transcriptional changes and chromatin rearrangement profiles, highlighting our previously reported importance of the ISGF3 complex in the IFN-γ response (Majoros et al., 2017; Platanitis et al., 2019). We further observe a significant gain in genome-wide chromatin accessibility at the TSSs of ISG in response to both stimuli, which strongly correlated with mRNA expression. An active promoter configuration is further underlined by the gain of active histone marks (H3K27ac) and the removal, or lack of deposition, of transcriptional silencing modifications (H3K27me3). Finally, we present a new model, which highlights the importance of the ISGF3 complex in priming the homeostatic chromatin landscape on ISG, as well as in remodeling the local chromatin structure downstream of both type I and type II IFN signaling.

## Results

### Interferons increase the A compartment strength at interferon-stimulated gene loci

To examine and compare the immediate-early response effects of IFN treatment on macrophage chromatin 3D conformation on a whole-genome level, we treated mouse bone marrow-derived macrophages (BMDM) for 2 h with either IFN-I or IFN-γ and analyzed chromatin contacts by Hi-C in two biological replicates.

First, we determined the reproducibility of our data with HiCRep, a framework using a stratum-adjusted correlation coefficient for quantifying sample similarity from Hi-C interaction matrices (Yang et al., 2017) (Figure S1A). The replicates clustered together according to their treatment, allowing us to merge replicates and thus obtain a higher coverage for subsequent analysis. In addition to the overall sample similarity, the data show that both IFN treatments produce changes in the overall chromatin structure.

Genome-wide analyses revealed that euchromatin and heterochromatin regions are spatially segregated (Dixon et al., 2012; Lieberman-Aiden et al., 2009; Rao et al., 2017). Thus, Hi-C pairwise interaction heatmaps not only show that individual active and inactive regions remain spatially separated but also that each type of chromatin also interacts with distal regions of the same type. Here we used eigenvector deconvolution analysis of our Hi-C data to identify regions that correspond to each type of chromatin. “A” compartments represent active regions that are usually defined as gene-rich with ongoing transcription and high accessibility. “B” compartments on the other hand are inactivated regions containing tightly packed and inaccessible chromatin. Integrating our previously published RNA-seq datasets with Hi-C experiments performed under matching experimental conditions highlighted a correlation between the compartment scores and the RNA-seq coverage, confirming both the segregation of the genome into A and B compartments based on transcriptional activity and the accuracy of our compartment analysis (Figures S1B–S1D) (Platanitis et al., 2019).

**Figure 1:**
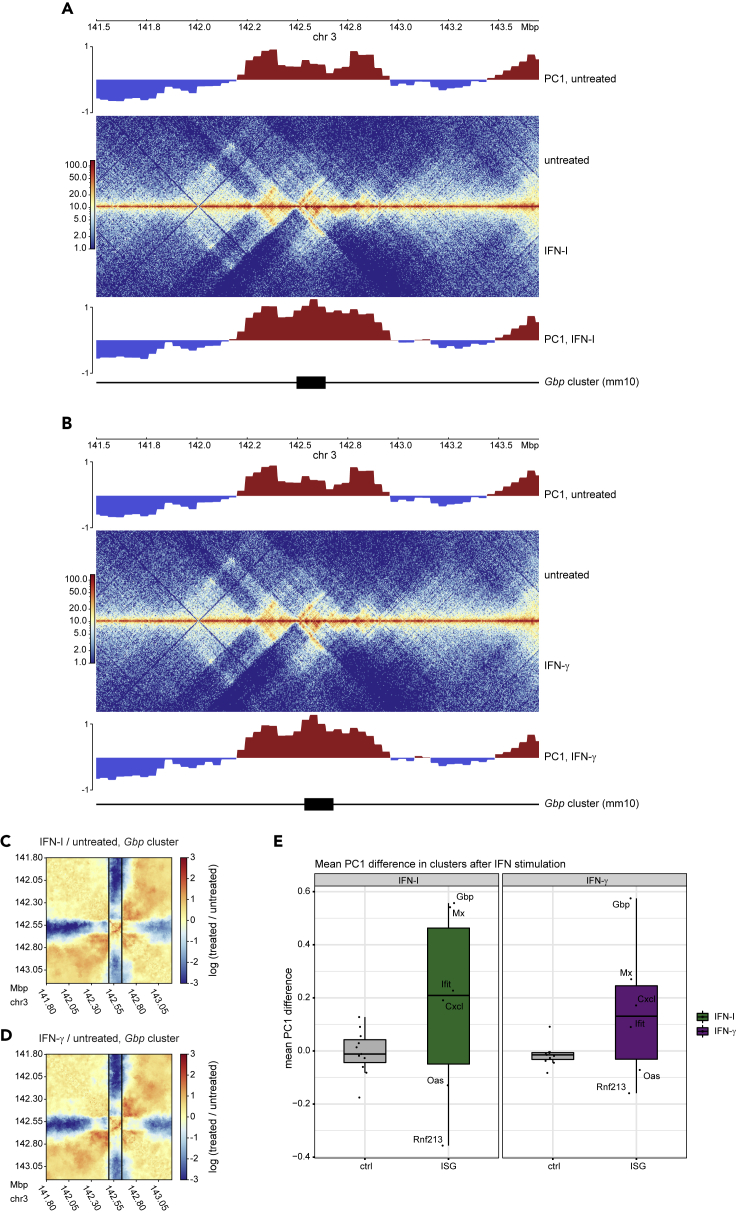
IFN treatment rearranges host cell genome 3D structure (A and B) Hi-C contact maps around the *Gbp* locus of untreated (upper middle) and A IFN-I treated (2h) or B IFN-γ treated (2h) (lower middle) primary murine bone marrow-derived macrophages (BMDM; merge of two replicates per condition). Upper and lower panel, PC1 values indicating compartments. (C and D**)** Ratio plots around the *Gbp* locus comparing contact maps of C IFN-I or D IFN-γ treated and untreated BMDM, generated using the flexible binning method Serpentine. Black vertical lines indicate the borders of the *Gbp* locus. Red regions indicate increased, blue regions decreased interaction after treatment with IFN. (E) Quantification of PC1 differences (40 kbp bins) at indicated ISG clusters in response to IFN-I or IFN-γ treatment (2h), as compared to control regions of the same sizes, located up- and downstream of the respective regions. Mean differences across all bins overlapping the respective regions are shown

A large fraction of ISG is organized in clusters on different chromosomes. Inspection of the contact maps in combination with the predicted compartment score (PC1; as derived from principal component analysis), revealed that the strongest effects within compartments occur in clustered ISG (Figures 1A and 1B). Of note, the *Gbp* gene cluster showed rearrangements in the contact map as well as an increase in compartment scores, resulting in a change in compartment strength on IFN-I and IFN-γ treatment (PC1 tracks). Additionally, we observed differences in contacts between the *Gbp* cluster and the surrounding regions on IFN treatment, with a decrease in contacts between the *Gbp* cluster and the neighboring regions (Figures 1C and 1D). The data obtained at the *Gbp* cluster were corroborated by a quantitative analysis of compartmentalization changes comparing ISG loci with size-matched control regions up- and downstream of ISG loci (Figure 1E, methods, Table S1). With the exception of *Oas* and *Rnf213*, the investigated ISG-associated regions showed an increase in A compartment strength that exceeded that of the control loci.

The lack of compartment switching demonstrates that the observed regions are already in an active state before IFN treatment. This observation is in line with previous findings showing that many ISG are already transcribed at low, basal levels in the absence of any stimulus (Gough et al., 2012; Platanitis et al., 2019). Yet, the change in compartment strength on IFN-I and IFN-γ treatment correlates with a higher transcriptional activity of the genes located within those compartments. Investigation of saddle plots indicated that on a genome-wide scale the interactions of A/B compartments are largely unaffected by short-term IFN-I and IFN-γ signaling (Figures S1E–S1G). This result is in line with previous studies showing that short-term exposure to other extracellular signals likewise has no effect on higher-order chromatin structures (Comoglio et al., 2018; Le Dily et al., 2014; Jin et al., 2013).

Taken together, we conclude that both IFN types, while reinforcing A compartments at ISG loci, do not globally change the genomic arrangement of compartments. This suggests that 3D alterations may result from changes in DNA loops.

### Type I interferon signaling elicits the rapid rearrangement of chromatin-chromatin interactions in interferon-stimulated gene-rich regions

To further compare the effects of each IFN type on the chromatin structure, we systematically analyzed differences in the contact maps and performed loop calling using the pattern detection algorithm chromosight (Matthey-Doret et al., 2020). Here, each detected loop is represented by its start and end position, as well as a Pearson correlation score. For visualization, the detected chromatin loops are represented as arcs, whose darkness of color increases proportionally to the loop scores. We first confirmed that single replicates show similar results in regard to the loop calling process at ISG loci, allowing us to merge the replicates for obtaining denser contact maps (Figures S2A–S2C).

**Figure 2:**
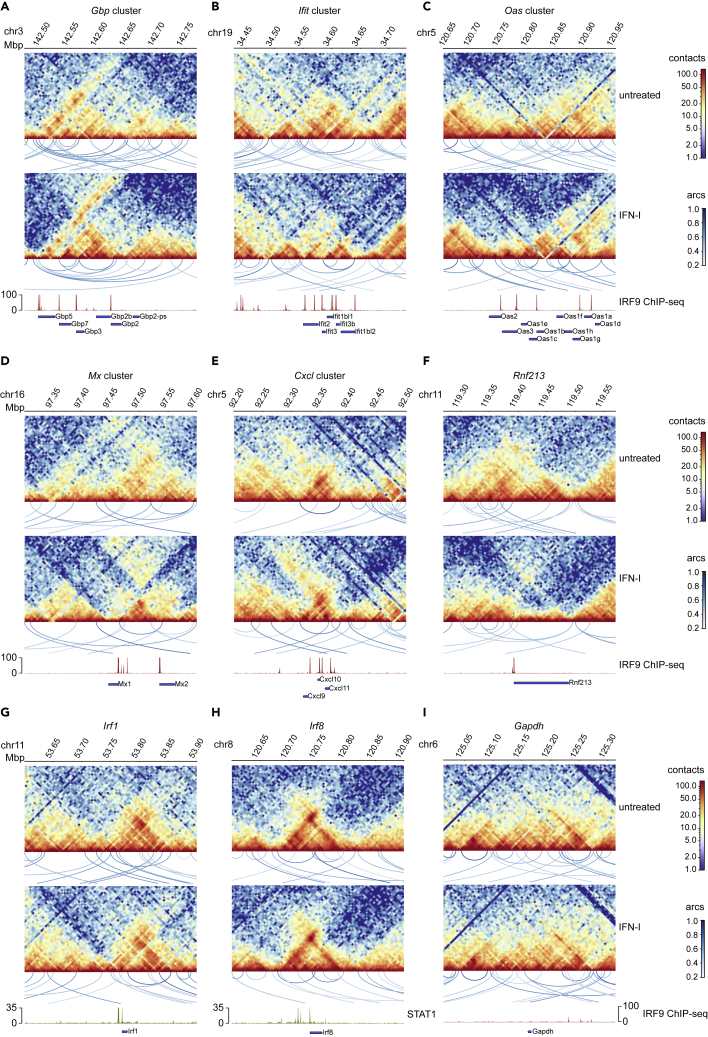
IFN-I treatment reshapes chromatin-chromatin contacts in ISG clusters (A–I) Hi-C contact maps (merge of two replicates per condition, log1p scale) of untreated and IFN-I treated (2h) BMDM. Visualization of pattern detection with chromosight (loops between two loci are indicated as arcs). Color coding of arcs corresponds to Pearson correlation scores. Loops with a score >0.35 are shown (maximum size/ylim = 500 kbp). Lower panel, IRF9/STAT1 ChIP-seq tracks after IFN-I (1.5h) stimulation and gene annotations are shown to visualize ISGF3/GAF binding sites. Only ISG of the corresponding gene clusters are visualized

We examined whether chromatin contacts within ISG loci are subject to IFN-induced changes by exploring the genomes of resting or IFN-I treated BMDM. Indeed, the resulting contact maps, representing a depth of about 500 Mio read pairs/contacts, revealed changes at all examined ISG clusters (Figures 2A–2F). Comparably small changes were observed at the *Irf1* and *Irf8* loci, both of which are not within ISG clusters and not strongly induced by IFN-I (Figures 2G and 2H). In regions where clear changes in the contact maps and compartment scores occurred, loop positions and scores were also affected. Alterations in these clusters represented predominantly increases in intra-cluster, short-range chromatin contacts. This effect was especially noticeable at the *Ifit* and *Oas* gene clusters (Figures 2B and 2C). Within the *Gbp* cluster on chromosome 3, short-range contacts between loci inside the region of interest showed an increased interaction after IFN-I treatment (Figure 2A). Additionally, many long-range contacts decreased in strength after treatment, consistent with the analysis presented in Figure 1C. Striking effects of IFN-I stimulation were also observed close to the end of chromosome 16, where *Mx1* and *Mx2* are located (Figure 2D). Here, we found that the treatment of the cells dramatically increased contacts in the area between the two genes. *Rnf213*, which is not embedded within a cluster, shows that short-range conformation changes even occur on single ISG loci (Figure 2F). Treatment with IFN-I did not notably change the loop structure of the *Gapdh* locus, confirming that the changes noted at ISG clusters are specific and caused by cytokine treatment (Figure 2I).

Taken together, these results demonstrate that chromatin rearrangements in response to IFN-I treatment can mostly, but not exclusively, be observed around regions containing larger numbers of ISG and some of the clusters show similar patterns characterized by increases in intra-cluster and a concomitant loss of long-range contacts.

### Type I interferon and interferon-γ show a high overlap of transcriptional profiles and 3D chromatin rearrangements

**Figure 3:**
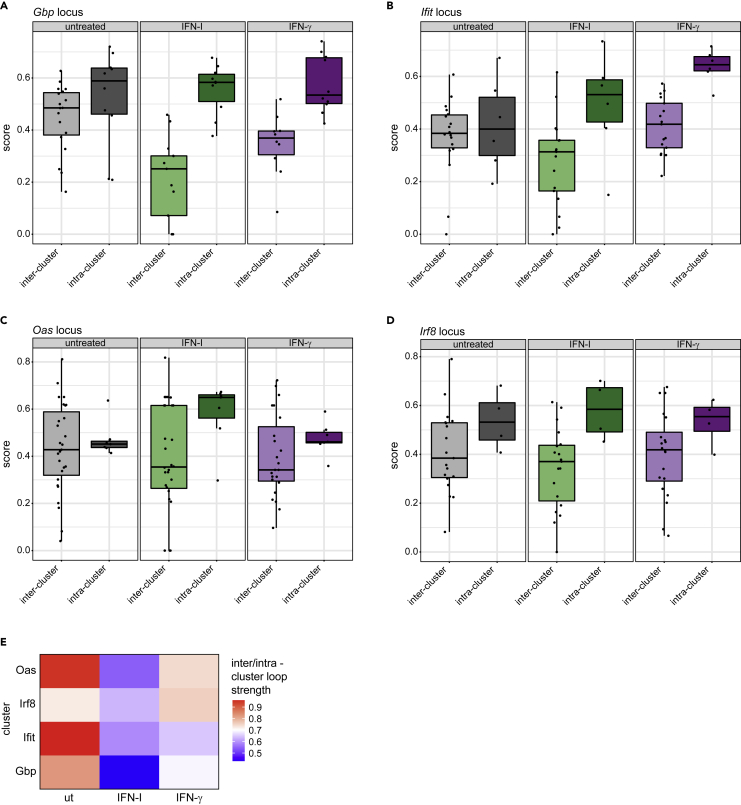
Effects of IFN-I and IFN-γ on loops within ISG clusters and with outside regions (A–D) Boxplots of inter- (one anchor in cluster region) and intra-cluster (both anchors in cluster region) loop scores for ISG loci in untreated (gray), IFN-I (2h, green) or IFN-γ (2h, purple) treated BMDM. (E) Heatmap summarizing the quantification of inter-/intra-cluster loop strength ratios. Ratios of median inter- and intra-cluster loop scores for each cluster untreated, IFN-I or IFN-γ treated BMDM are shown

When comparing IFN-I to IFN-γ treatment, we realized that both cytokines triggered very similar 3D chromatin changes at the majority of ISG clusters which are characterized by a prevalence of ISRE sequences (Figures S3A–S3D, Table S2). In contrast, *Irf1*, an ISG carrying only a GAS element, and thus a STAT1 binding site within its proximal promoter (Pine et al., 1994; Rein et al., 1994), showed stronger changes in its contact map when cells were treated with IFN-γ (Figures 2G and S3E). The *Irf8* gene which is similarly regulated by STAT1 homodimers (Kanno et al., 1993; Parrini et al., 2018) showed very little change in loop structure, in line with the much weaker inducibility of the *Irf8* gene compared to *Irf1* (Figure 2H, Table S1). To more systematically assess the changes in the chromatin loop structure across these loci, the ratio between the overall strength of inter-vs intra-cluster loops was quantified. This analysis was limited to loci containing a sufficiently high number of loops within the regions of interest (see STAR Methods: inter-/intracluster loop strength ratio calculations). We then compared the results at the ISG clusters (showing strong IFN-induced changes) to the *Irf8* locus, which shows a limited response to IFN treatment (Figures 3A–3E). The resulting ratios (Figure 3E) are consistent with the visual representation of loops provided in Figures 2A–2F and our interpretation of a relative increase in intra-cluster loops formation. Whereas the dominant effect causing this ratio shift was an increase in intra-cluster loop strength at the *Ifit* and *Oas* clusters (Figures 3A and 3C), loss of long-range interactions contributed more to the ratio change at the *Gbp* locus (Figure 3D).

**Figure 4:**
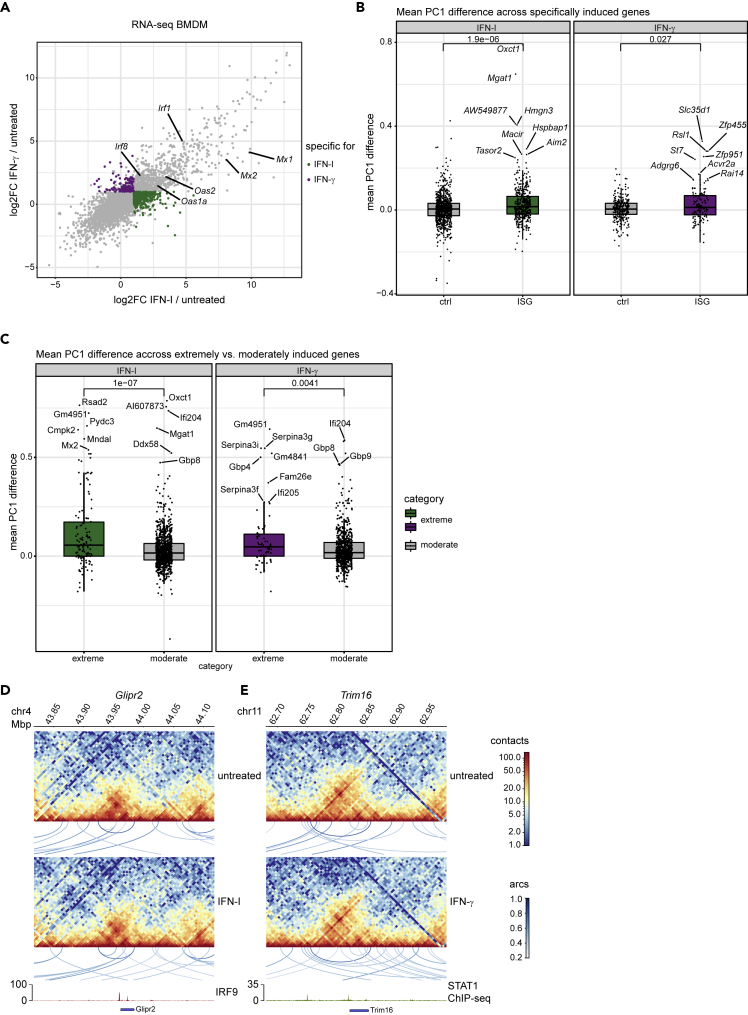
Inducibility by interferons correlates with changes in A compartment strength (A) Scatterplot comparing log2-fold-changes (log2FC) in mRNA abundances from IFN-I (2h) and IFN-γ (2h) treated BMDM. Genes significantly up-regulated (log2FC ≥ 1, adjusted p value ≤ 0.05) in one, but not the other treatment (log2FC < 1) are colored in green (IFN-I) and purple (IFN-γ), respectively. (B) Differences in PC1 (as described in Figure 1E) after treatment with IFN-I or IFN-γ (2h) at gene loci, specifically induced by IFN-I or IFN-γ (2h) in RNA-seq. Mean values of PC1 differences for bins overlapping each gene region are plotted. For each gene, two control regions of the same size, respectively 1 Mb up- and downstream of the gene, are considered (p = 1.9 × 10^−6^ for IFN-I, p = 0.027 for IFN-γ, Wilcoxon rank-sum test for unpaired data). (C) Similar to B, for gene groups that show extreme (log2FC > 5) or moderate (log2FC 1-5) induction in RNA-seq in response to the respective IFN stimuli (IFN-I or IFN-γ, 2h; p = 1,0 × 10^−7^ for IFN-I; p = 0.0041 for IFN-γ, Wilcoxon rank-sum test for unpaired data). (D and E) Upper and middle panels: Hi-C contact maps (merge of two replicates per condition, log1p scale) of untreated and IFN-I or IFN-γ-treated (2h) BMDM. Visualization of pattern detection with chromosight (loops between two loci are indicated as arcs). Arcs are color-coded according to their Pearson correlation scores. Loops with a score >0.35 are shown (maximum size/ylim = 500 kbp). Lower panel: IRF9/STAT1 ChIP-seq tracks after the respective treatment (1.5h) and gene annotations are shown to visualize ISGF3/GAF binding sites

Although evidence that STAT binding initiates loop formation is currently elusive, the stronger change in loop formation after IFN-γ treatment at the *Irf1* gene (Figure S3E) is in agreement with the paradigm of IFN signaling, which posits a larger contribution of STAT1 homodimers to IFN-γ-induced transcription when compared to those produced by IFN-I. On the other hand, loop formation at ISG clusters is in line with our previous notion that ISGF3 activity carries significant weight in the entirety of IFN-γ-induced transcriptome changes. To further address differences and similarities, we directly compared the early transcriptional response to both IFN types in BMDM based on RNA-seq datasets previously generated in our lab. Surprisingly, many genes and especially the strongly induced ones were affected in a similar fashion by IFN-I and IFN-γ (Figure 4A, Table S3). However, groups of genes displaying weaker but significant changes showed preferences for IFN-I or IFN-γ (Figure 4A; purple and green dots, respectively). Overall, this finding is in agreement with similar chromatin rearrangements produced by both IFN types at many ISG-rich regions. At the same time, it supports the previously reported role of the ISGF3 complex in the IFN-γ response (Majoros et al., 2017; Platanitis et al., 2019; Rauch et al., 2015). To better characterize gene loci responding exclusively to one IFN type only, we first performed compartment strength quantification, revealing that increases in A compartment strength were minimal in a large proportion of these genes (Figure 4B), possibly owing to their low inducibility. A substantial part of strongly and even moderately induced ISGs, on the other hand, showed larger increases, with the loci bearing the most strongly increased genes showing larger increase of A compartment strength than those showing a moderate induction (Figure 4C). On average, IFN-I produced larger increases in A compartment strength than IFN-γ. We also investigated the chromatin loop structure at the loci of the *Glipr2* and *Trim16* genes that respond specifically to IFN-I and IFN-γ, respectively. In line with their low inducibility, we found their chromatin loop structure to be virtually unchanged by IFN treatment (Figures 4D and 4E).

### IFN-stimulated gene factor 3 regulates chromatin accessibility at interferon-stimulated gene loci in homeostatic and IFN-induced conditions

A hallmark of the immune system is its ability to quickly transition between resting and stimulated states. Recent studies have shown large-scale chromatin remodeling in cells responding to immune stimuli (Calderon et al., 2019; Ostuni et al., 2013). In order to complement our Hi-C data with information about chromatin accessibility, we performed ATAC-seq, designing experiments to reveal the impact of both IFN types on nucleosome structure. All ATAC-seq samples were generated in three biological replicates and a high correlation between replicates was obtained (Figure S4A).

To gain an overview of the rearrangements occurring at the loci showing the strongest transcriptional induction, we first divided the genome according to the top 200 IFN-I or IFN-γ -induced genes and the remaining ones (non-ISG), using our previously published RNA-seq analysis (Platanitis et al., 2019). In light of our recent data showing the role of ISGF3 complexes in both homeostatic and induced ISG expression, we included *Irf9−/−* macrophages. These cells reveal the impact of ISGF3. Of note, the acronym ISGF3 describes two related complexes: the full version formed by STAT1, STAT2, and IRF9 dominating the IFN response, as well as the “light” version formed by STAT2 and IRF9, whose main importance lies in the activation of homeostatic ISG transcription (Platanitis et al., 2019).

**Figure 5:**
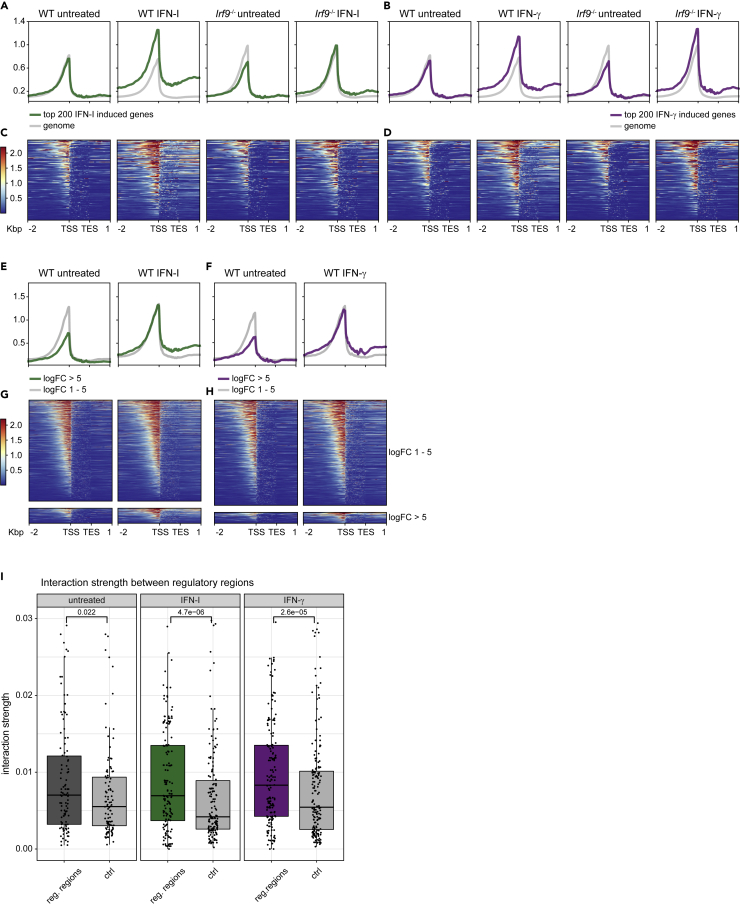
ISGF3 complex-dependent effects of IFN signaling on chromatin accessibility (A and B) Summary profile plots of chromatin accessibility in BMDM generated by using normalized read coverages for untreated and IFN-I/IFN-γ treated wildtype and *Irf9−/−* BMDM as indicated. The colored profile is an average of the top 200 genes (log2FC) upregulated after IFN-I (2h; green profile) and IFN-γ (2h; purple profile) treatment, respectively, according to RNA-seq. The gray profile is an average of the respective remaining gene regions. (C and D) Heatmaps of chromatin accessibility in regions of the top 200 genes (log2FC) upregulated after IFN-I (2h) and IFN-γ (2h) treatment, respectively, according to RNA-seq. (E and F) Similar to A, B. The colored profile is an average of genes induced by the respective treatments with a log2FC > 5, the gray profile indicates an average of genes induced with a log2FC of 1-5. (G and H) Similar to C, D, but considering gene groups shown in E, F, respectively. Profiles and heatmaps in A-H are shown for the regions of the genes, including 2 kbp upstream of the TSS and 1kbp downstream of the TES as indicated below the plots. Shown are merged data from three biological replicates (See also Figure S4). (I) Interaction strength (as inferred from normalized Hi-C matrices; y axis limit 0.03) between regulatory regions overlapping the ISG clusters shown in Figure 2. Signals from untreated, IFN-I (2h) and IFN-γ (2h) treated BMDM are shown. All pair-wise interactions between regulatory regions within the same clusters are considered. Control pairs indicate the interaction between regulatory regions and other, non-regulatory regions

Chromatin at non-ISG did not undergo major changes upon treatment with either IFN type (Figures 5A, 5B, S4B, and S4C, gray profile, large lower panels). Importantly, the loss of IRF9 produced an accessibility decrease in a subset of ISG loci during cell homeostasis, which is consistent with the role of STAT2/IRF9 in constitutive ISG expression (Figure 5). IFN-I stimulation was marked by an increase in the number and intensity of ATAC-seq signals at ISG loci, which was strongly reduced in *Irf9−/−* macrophages (Figures 5A and 5C). IFN-γ treatment also increased the accessibility at ISG loci; however, loss of IRF9 did not markedly reduce the accessibility in this subset of genes (Figures 5B and 5D). We then dissected the dependency of the accessibility changes in the level of ISG inducibility as determined by RNA-seq (Figures 5E–5H). This produced the unexpected result that highly and moderately induced ISG showed similar levels of accessibility after IFN treatment. However, highly induced genes were less accessible than moderately induced genes when compared in resting cells. This might be explained by tighter control of highly inducible genes against aberrant expression. Consistent with the compartment analysis, the group of genes with weak, but IFN type-specific inducibility did not undergo detectable changes in chromatin accessibility (Figures S4D and S4E).

ISG clusters contain multiple promoters with IFN response elements (see ChIP-seq tracks in Figures 2 and S2). The increased accessibility of these regions after stimulation with IFN suggests the possibility that this is structurally linked to the IFN-induced increase of the intra-vs inter-cluster chromatin loops (Figure 3). We explicitly tested this hypothesis by the integration of ATAC-seq and Hi-C data with the aim of quantifying interaction strengths between accessible regions and comparing them with sets of control regions of the same extension (for details see Materials and methods). In agreement with our hypothesis of preferential interactions between intra-cluster regulatory elements, accessible regions showed significantly larger interaction strengths compared to control regions in untreated cells (Figure 5I, Table S4). The significance of this difference further increases upon treatment with both IFN types.

**Figure 6:**
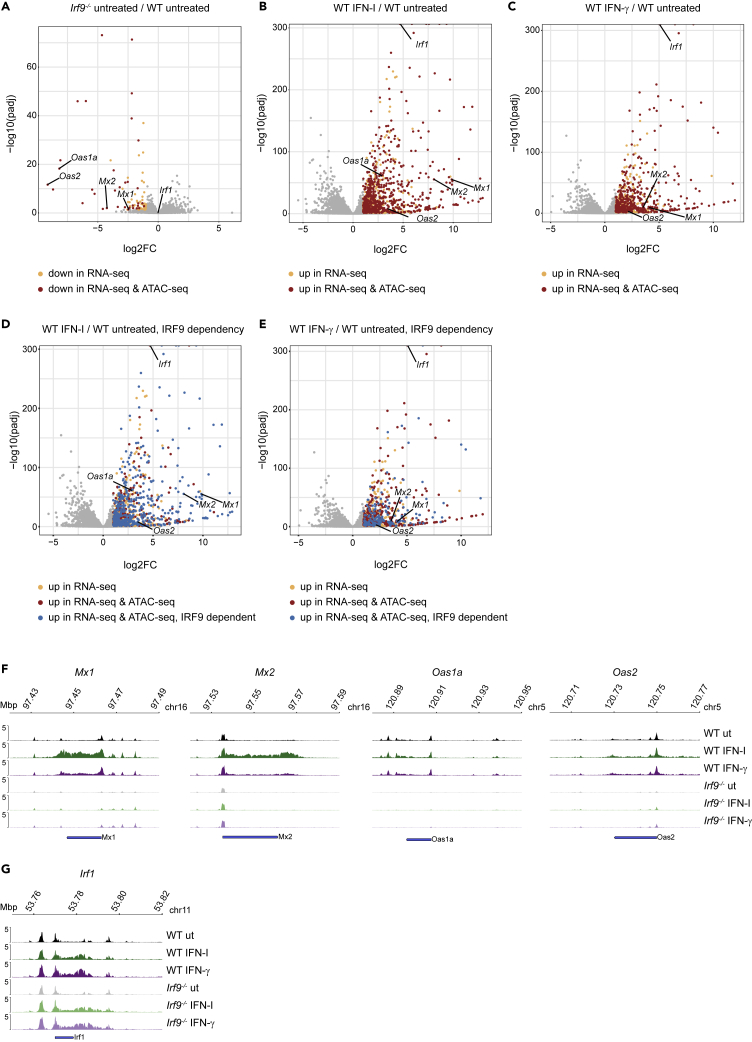
ISGF3 complex-dependent chromatin opening at ISG loci in homeostatic and interferon-induced states (A–F) Volcano plots of mRNA expression in BMDM in respective conditions. Each dot represents a gene. The log2-transformed fold change and -log10-transformed adjusted p values for gene expression are shown in x and y axis, respectively. (A) *Irf9*−/− vs wildtype BMDM in homeostatic condition: yellow dots represent genes significantly downregulated in *Irf9*−/− according to RNA-seq (log2FC ≤ 1, adjusted p value ≤ 0.05) and red dots represent genes which in addition showed significantly decreased accessibility at their respective loci according to ATAC-seq in *Irf9*−/− BMDM (log2FC ≤ 1, adjusted p value ≤ 0.05). (B) IFN-I (2h) stimulated vs untreated BMDM; **C** IFN-γ (2h) stimulated vs untreated BMDM. In the panels, yellow dots represent the genes that were significantly upregulated after either IFN treatment (log2FC ≥ 1, adjusted p value ≤ 0.05) according to RNA-seq. The red dots represent genes which in addition showed significant increase in chromatin accessibility (log2FC ≥ 1, adjusted p value ≤ 0.05) after corresponding IFN treatment at their genomic loci according to ATAC-seq data. (D and E) The blue dots represent genes which in addition, showed a significant decrease in chromatin accessibility at their genomic loci in *Irf9*−/− BMDM in response to 2h of either IFN treatment (ATAC-seq peaks, log2FC ≤ −1, adjusted p value ≤ 0.05). (F and G) Genome browser tracks for ATAC-seq at respective gene loci in wildtype and *Irf9*−/− BMDM treated with IFN-I or IFN-γ for 2h (one representative replicate). Data in A-E are derived from three biological replicates

In order to further investigate the ISGF3-dependent expression and the corresponding accessibility changes in IFN-treated cells, we integrated RNA-seq and ATAC-seq data from wildtype (wt) and *Irf9−/−* macrophages. As previously published, basal expression of a subset of ISG dropped in *Irf9−/−* macrophages (Figure 6A, colored dots, Table S5). These genes were further analyzed for corresponding changes in chromatin opening (red dots). *Oas1a, Oas2, Mx2,* and *Mx1* were among the genes, that showed a drop in both expression and accessibility. *Irf1*, a target gene requiring STAT1 homodimer activity, was not affected by the loss of IRF9. Furthermore, we noted a high degree of overlap between genes whose expression was induced by IFN-I and IFN-γ (Figures 6B and 6C; all colored dots, Tables S6 and S7) and genes showing increased accessibility by the respective treatment (red dots). For the majority of gene loci, the type I IFN-dependent increase in chromatin accessibility required the presence of ISGF3 (472 blue dots out of 680 red dots in Figure 6D, Table S6). By comparison, a significantly smaller fraction of loci with increased accessibility after IFN-γ stimulation was affected by the loss of IRF9 (153 blue dots out of 457 red dots in Figure 6E, Table S7; p = 2.2 × 10^−16^, Chi-squared test). Among the ISGF3-dependent genes were typical core ISG from the *Mx* and *Oas* families (Figures 6D–6F). By contrast, *Irf1* accessibility at and upstream of the TSS was only weakly affected by IFN and not at all by the loss of IRF9 (Figure 6G) (Platanitis et al., 2019). Taken together, our data support a view in which a subset of genes that are transcriptionally controlled by ISGF3 exhibit ISGF3-dependent chromatin accessibility at their genomic loci in both homeostatic and IFN-induced conditions.

### IFN-stimulated gene factor 3 controls the deposition of active histone marks and transcriptional activation at a subset of interferon-stimulated genes

The changes in macrophage genome structure occurring in response to challenges such as an infection ensure accessibility of critical regulatory DNA, thus allowing transcription factor binding and transcription of proinflammatory genes (Ostuni et al., 2013). An additional layer of gene control is formed by writers and erasers of histone marks.

We determined changes in activating or repressive histone marks with the aim of establishing whether both histone remodeling and modification at ISG promoters require ISGF3. To this end, we mined our IRF9 and RNA polymerase II (Pol II) ChIP-seq data as well as a publicly available H3K27ac ChIP-seq dataset (Mancino et al., 2015; Platanitis et al., 2019; Wienerroither et al., 2015). In addition, we performed site-directed ChIP using antibodies against H3K27ac, H3K27me3, and IRF9 in resting and IFN treated wt and *Irf9−/−* macrophages. IRF9 binding at *Mx1* and *Mx2* promoters is shown as a positive control (Figure S5).

**Figure 7:**
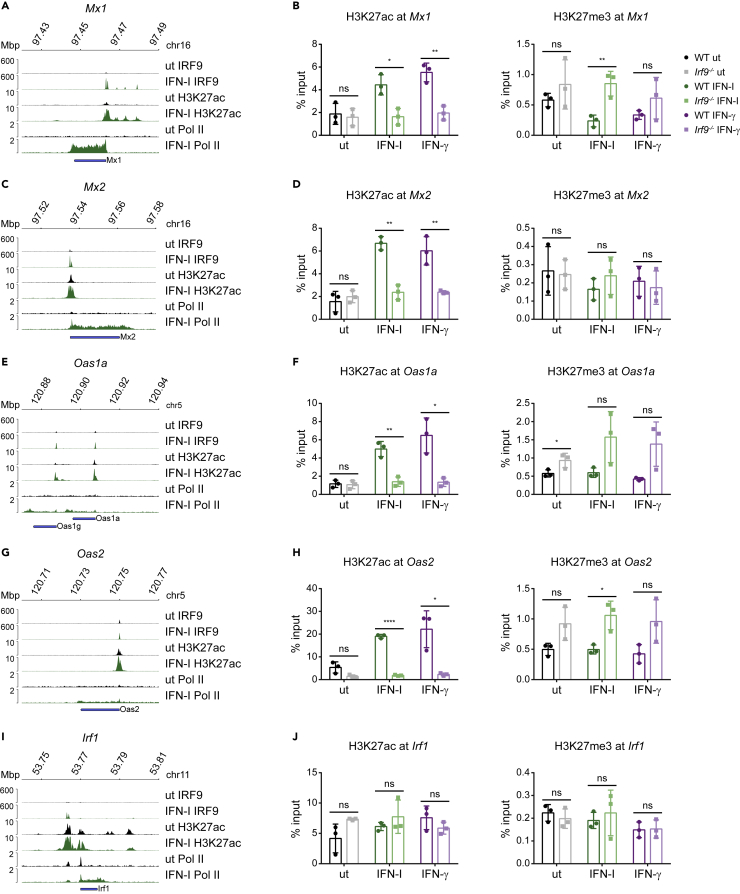
Histone modifications at ISG promoters correlate with the local chromatin accessibility (A, C, E, G, and I) Genome browser tracks representing published ChIP-seq datasets in BMDM with antibodies against the following proteins after IFN-I stimulation: IRF9 (1.5h), H3K27ac (1h) and Pol II (2h) showing promoter occupancy at indicated gene loci (one representative replicate). (B, D, F, H, and J) Site-directed ChIPs (*Mx1, Mx2, Oas1a, Oas2,* and *Irf1*) were performed in bone marrow-derived macrophages isolated from wildtype and *Irf9*−/− BMDM, treated for 2h either with IFN-I or IFN-γ and processed for ChIP with antibodies targeting H3K27ac and H3K27me3 as indicated (See also Figure S5). Data represent mean ± SD of biological triplicates, analyzed for statistical significance by Student’s two-tailed, unpaired t-test using Graph Pad Prism software. ∗p < 0.05; ∗∗p < 0.01; ∗∗∗p < 0.001

In line with previous observations (Platanitis et al., 2019), we noted different degrees of IRF9 pre-association with the promoters of the *Mx1, Mx2, Oas1a,* and *Oas2* genes in resting cells (Figures 7A, 7C, 7E, and 7G). This correlates with reduced chromatin accessibility in resting *Irf9−/−* BMDM (Figure 6A) and agrees with our recent finding showing that the STAT2-IRF9 complex controls basal ISG expression. The presence of H3K27ac was enriched at *Mx1, Mx2, Oas1a,* and *Oas2* gene promoters in response to both type I and type II IFN stimulation in wild-type cells and binding was significantly reduced in *Irf9−/−* BMDMs (Figures 7B, 7D, 7F, and 7H). In contrast, H3K27me3 was mostly unchanged in response to IFN-treatment. All promoters except that of *Mx2* showed a trend toward increased methylation in *Irf9−/−* macrophages which reached significance in case of the Oas2 and Mx1 genes after IFN-I-treatment. Hence, an IFN-independent, homeostatic ISGF3 activity is critically involved in removing repressive marks.

The pattern of H3K27ac deposition and Pol II loading was highly correlated with the promoter accessibility of the selected genes (Figures 7A, 7C, 7E, 7G, and 7I). Notably, at the *Irf1* gene, whose promoter is constitutively accessible independently of IFN-stimulation and the ISGF3 complex, the histone modifications did not change significantly after IFN treatment or upon IRF9 deficiency (Figures 7I and 7J). In accordance with recent findings (Steinparzer et al., 2019), this was also the only examined ISG with a preloaded Pol II.

Collectively, our findings demonstrate an ISGF3-complex dependent promoter activation in response to both IFN-I and IFN-γ, leading to increased chromatin accessibility, deposition of active histone marks, and transcriptional activation at a subset of ISG.

## Discussion

3D chromatin structure is vital for both the organization and function of our genomes. Mitosis most likely represents the biggest challenge to the proper folding and positioning of chromatin in the nuclei of dividing cells, but 3D organization is of similar importance also for gene expression, that is, the spatial arrangement of genes in active or inactive compartments and the controlled juxtaposition of regulatory elements in the process of transcriptional activation (Dekker and Mirny, 2016). Here, we investigated JAK-STAT signaling and its rapid control of nuclear ISG responses. We show that the structure of ISG loci undergoes similarly rapid changes: a reorganization of their loop formation and remodeling of nucleosomes that increases the accessibility of the DNA containing regulatory elements. These changes are accompanied by the establishment of activating histone marks and, at most loci, the *de novo* recruitment of Pol II. Our studies are a starting point for future investigations into the precise mechanisms of chromatin reorganization at ISG loci.

The contact maps and the inferred loops established from our Hi-C analyses show clustered ISG within the same TAD. This suggests that the chromatin loops at these loci may form and rearrange in accordance with the loop extrusion model, the assembly of cohesin/NIPBL/MAU2 complexes at binding sites for transcription factor CTCF, and the subsequent ATP-dependent extrusion of DNA loops (Davidson et al., 2019; Kim et al., 2019; Thiecke et al., 2020; Wutz et al., 2017, 2020). The size of such loops is determined by the cohesin release factor WAPL (Haarhuis et al., 2017). WAPL repression by transcription factors may influence loop extrusion, as has been demonstrated for Pax five at the Ig heavy chain locus (Hill et al., 2020). The loop extrusion model thus provides an arena for further studies of the mechanisms by which IFN rearrange the chromatin of ISG loci. It is in agreement with a recent report about the importance of cohesin in the transcriptional response of macrophages to LPS (Cuartero et al., 2018).

While mechanistic implications of IFN-induced or disrupted loops require further investigation, inducible changes in 3D chromatin structure are corroborated by the consistent changes in the loop interaction strength within ISG clusters (Figure 1) and the integrative analysis of the Hi-C pattern in the context of chromatin accessibility at key ISG (Figure 3). These data are in line with the notion that clusters containing many ISG and their ISRE-containing promoters, adopt 3D arrangements that bring the regulatory elements in the region in close proximity, particularly after IFN treatment. This may in turn increase the local concentration of ISGF3 complexes with the ability to exert activity upon all genes in the cluster. Validating this hypothesis will involve the editing of ISRE sequences in their genomic context and the study of the resulting changes in transcriptional activation and the 3D chromatin structure of the corresponding ISG locus.

Previous studies addressing chromatin remodeling at certain ISG loci conclude that nucleosome rearrangements may occur either before or after stimulation with IFN (Cheng et al., 2019; Mostafavi et al., 2016; Ni et al., 2005). Our ATAC-seq data now demonstrate on a genome-wide scale that IFN treatment opens chromatin at a large fraction of ISG promoters, but that some were rendered accessible independently of IFN. In addition, the results in IRF9-deficient cells clearly show that ISGF3 complexes are part of the machinery for chromatin remodeling at ISG. However, this is again not generally the case for all ISG. Thus, ISGF3 may form complexes with and help to recruit chromatin remodelers as has been suggested (Au-Yeung and Horvath, 2018; Gnatovskiy et al., 2013). On the other hand, some genes manage to open their promoter chromatin via ISGF3-independent mechanisms both during homeostasis and after exposure to IFN. Generally speaking, ISG fall into the four categories: (i) homeostatic accessibility, ISGF3-dependent, (ii) homeostatic accessibility, ISGF3-independent, (iii) IFN-induced accessibility, ISGF3-dependent, and (iv) IFN-induced accessibility, ISGF3-independent. We also noted that open chromatin at gene bodies frequently correlates with ongoing ISG transcription. This observation agrees with a report in virus-infected cells showing transcription-dependent chromatin rearrangements in influenza virus-infected cells (Heinz et al., 2018).

Given the distinct activities of the two IFN types in the immune system, the degree of overlap between IFN-I and IFN-γ-induced transcriptomes is unexpected. To our surprise, similarities between the molecular mechanisms of transcriptional control by the two cytokines extended to all levels of regulation, from 3D loop rearrangement and promoter accessibility to histone modification and Pol II recruitment. In part, this may reflect the activity of the ISGF3 complex which shows the expected importance in gene regulation by IFN-I, but a larger than expected impact on IFN-γ-induced gene control. Compared to IFN-I signaling, STAT2 tyrosine phosphorylation downstream of the IFN-γ receptor is much less pronounced (Ho et al., 2016; Platanitis et al., 2019). In fact, the unphosphorylated fraction of STAT2 was shown to block nuclear entry of phosphorylated STAT1 through the formation of hemi-phosphorylated heterodimers and thus dampen the response to IFN-γ (Ho et al., 2016). In light of these findings it is surprising that most ISG responding to both IFN types show strong, IFN-γ-induced association with ISGF3 by ChIP-seq (Platanitis et al., 2019) and, consistently, a critical requirement for ISGF3 in producing open chromatin, depositing active histone marks and recruiting Pol II. One possible explanation for the discrepancy between little cytoplasmic STAT2 phosphorylation and the comparably large availability of nuclear ISGF3 for gene regulation could be trapping of nuclear complexes either by unspecific interaction with DNA, chromatin proteins, or the nuclear matrix. Trapping of ISGF3 may again increase its local concentration at specific locations of the genome.

Why then is the immunological activity of IFN-γ so different from that of IFN-I, particularly in macrophages? An important explanation may be provided by the rather small group of ISGF3-independent early response genes such as *Irf1*. *Irf1* is a first-tier gene encoding a transcription factor with a large contribution to the secondary IFN-γ regulome (Langlais et al., 2016; Ramsauer et al., 2007). This differs from IFN-I-induced genes which are largely independent of secondary IRF1 effects (Forero et al., 2019). Consistent with the notion that the *Irf1* gene is a primary response gene to IFN that is regulated independently of ISGF3, its chromatin loop structure, promoter chromatin opening, the deposition of activating marks and the recruitment of Pol II are largely preset under homeostatic conditions. This situation is reminiscent of primary, LPS-induced genes that are regulated at the level of transcription elongation (Adelman et al., 2009). The group of secondary, IRF1-dependent IFN-γ target genes includes antimicrobial effector genes such as *Nos2* (*iNos*) and contributes to the macrophage-activating properties of IFN-γ (Langlais et al., 2016). IRF8 is another STAT1 homodimer-dependent gene and important regulator of the IFN-γ-induced transcriptome in myeloid cells (Langlais et al., 2016; Platanitis and Decker, 2018). Its induction by IFN is much weaker than that of IRF1 but it plays an important role in the acquisition of transcriptional permissiveness at IFN-responsive enhancers (Mancino et al., 2015; Platanitis and Decker, 2018). According to our dataset the *Irf8* locus shows the same IFN/ISGF3 independence in chromatin accessibility as the *Irf1* locus and, similar to the Irf1 locus, its chromatin loop structure is pre-determined and largely unchanged by IFN signaling. Thus, while there is clearly a large overlap and shared ISGF3-dependent regulome between IFN-I and IFN-γ that is responsible for common biological activities such as the antiviral state or regulation of inflammation, STAT1 homodimers, as drivers of IRF1 and IRF8 synthesis, are critical determinants for the role of IFN-γ as a macrophage-activating factor. Although our study was focused on the immediate response to IFN-I and IFN-γ, delayed stages of the response to the cytokines are most likely characterized by larger differences between their induced transcriptomes, owing to a different deployment of transcription factors like IRF1 that determine secondary gene induction. Furthermore, IFN-γ reportedly promotes macrophage activation by selective repression of LPS-induced genes (Kang et al., 2019).Thus, the interplay and integration of the different levels of gene control downstream of the IFN-induced JAK-STAT pathways will remain an interesting topic for future studies.

### Limitations of the study

The data reported here address mechanisms of gene control and transcriptome changes in the immediate response of macrophages to IFN. They show a large degree of overlap between IFN-I and IFN-γ with regard to these parameters. As pointed out in the discussion of our data, this large degree of similarity may decrease through the deployment of higher order responses at later stages. In future studies, it will be necessary to extend the current approach to delayed phases of the response to IFN. Although the failure to detect 3D changes at non-clustered loci that are only weakly inducible may indicate a preset chromatin organization, it may also be a reflection of the limited sensitivity of the Hi-C technique at the read depth employed in our study. Further insight as to the mechanism controlling increases in intra-cluster loops will result from investigations in IRF9-deficient macrophages and from the deletion of regulatory DNA elements from their promoter context. Finally, it will be of interest to determine the extent to which our results can be extrapolated to other macrophage populations or cell types.

## STAR★Methods

### Key resources table

**Table:** 

REAGENT or RESOURCE	SOURCE	IDENTIFIER
**Antibodies**		

anti-H3K27Ac	Cell Signaling	Cat# 8173; RRID:AB_10949503
anti-H3K27me3	Active Motif	Cat# 39155; RRID:AB_2561020
Anti-IRF9	6F1-H5 supernatant	Platanitis et al., 2019

**Chemicals, peptides, and recombinant proteins**		

mu recIFN-β	PBL Assay Science	Cat# 12400-1
mu recIFN-γ	A kind gift from G. Adolf, Boehringer Ingelheim, Vienna	n/a
hu recCSF-1	A kind gift from L. Ziegler- Heitbrock, Helmholtz Center, Munich, Germany	n/a
Tn5 transposase	Illumina	Cat# 15027865

**Critical commercial assays**		

Magnetic Dynabeads protein G	Life Technologies	Cat# 10003D
MBSpure beads	Vienna Biocenter NGS Facility	https://www.viennabiocenter.org/vbcf/next-generation-sequencing/
MinElute Columns	Quiagen	Cat# 28006
PCR master mix	NEB	Cat# M0492L
Arima-Hi-C kit	Arima Genomics	P/N Cat# A510008
Ultra II DNA library prep	NEB	Cat# E7103S

**Deposited data**		

Hi-C and ATAC-seq data	this paper	SRA BioProject: PRJNA694816
IRF9 ChIP-seq	Platanitis et al., 2019	GEO: GSE115435
STAT1 ChIP-seq	Platanitis et al., 2019	GEO: GSE115435
Pol II ChIP-seq	Wienerroither et al., 2015	ArrayExpress: E-MTAB-2972
RNA-seq	Platanitis et al., 2019	GEO: GSE115435

**Experimental models: Organisms/Strains**		

Model organism: mouse C57BL/6N	Janvier Labs	https://www.janvier-labs.com/en/fiche_produit/c57bl-6nrj_mouse/
Model organism: mouse C57BL/6N IRF9-/-	University of Veterinary Medicine, Vienna	Kimura et al., 1996

**Oligonucleotides**		

Primers for ChIP-Q-PCR	n/a	Table S5

**Software and algorithms**		

Hi-C data processing	https://zenodo.org/record/2669513#.YA8UvWRKjZk; (Ewels et al., 2020)	Nf-core/Hi-C v1.1.0
Hi-C data sample similarity	https://github.com/cmdoret/hicreppy	HiCreppy v0.0.6
Hi-C data PCA/Compartment analysis	https://cooltools.readthedocs.io/en/latest/index.html	Cooltools v0.3.0
Hi-C data ratio plots	Baudry et al., 2020	Serpentine
Hi-C data pattern detection, reproducibility, loop strength ratio calculations	Matthey-Doret et al., 2020	Chromosight v1.3.3
ATAC-seq data processing	https://zenodo.org/record/3965985#.YA8a8GRKjZk	nf-core/atacseq v1.2.1
ATAC-seq heatmaps	https://zenodo.org/record/3965985#.YA8XPGRKjZk	Deeptools v3.4.3
ATAC-seq/RNA-seq integration	https://bioconductor.org/packages/release/bioc/htm l/DESeq2.html	DESeq2 v1.16.1
ATAC-seq/RNA-seq integration	Heinz et al., 2010	HOMER script *annotatePeaks.pl*.
Statistical analysis ChIP-Q-PCR	www.graphpad.com	GraphPad Prism

### Resource availability

#### Lead contact

Further information and requests for reagents may be directed to, and will be fulfilled by, the lead contact, Thomas Decker (thomas.decker@univie.ac.at).

#### Materials availability

This study did not generate new unique reagents.

### Experimental model and subject details

#### Animals

C57BL/6N mice (wildtype, *wt*) were purchased from Janvier Labs. *Irf9-/-* mice (Kimura et al., 1996) were backcrossed for more than 10 generations on a C57BL/6N background. Mice were housed under identical conditions in a specific-pathogen-free (SPF) facility according to the Federation of European Laboratory Animal Science Association (FELASA) guidelines and additionally monitored for being norovirus negative. Mice were bred under the approval of the institutional ethics and animal welfare committee of the University of Veterinary Medicine of Vienna and the national authority Federal Ministry Republic of Austria Education Science and Research section 8ff of the Animal Science and Experiments Act (Tierversuchsgesetz [TVG], BMWF-68.205/0068-WF/V/3b/2015 and GZ 2020-0.200.397). The study did not involve animal experiments as defined in the TVG and did not require ethical approval according to the local and national guidelines. Female mice were used at an age of 8-12 weeks for the isolation of bone marrow.

### Method details

#### Isolation, culture and cytokine treatment of bone marrow-derived macrophages

Bone marrow-derived macrophages (BMDM) were differentiated from bone marrow isolated from mouse femurs and tibias. The bones were flushed with Dulbecco’s modified Eagle’s medium (DMEM) (Sigma-Aldrich) and cells were differentiated in DMEM containing recombinant M-CSF (a kind gift from L. Ziegler- Heitbrock, Helmholtz Center, Munich, Germany). BMDM were stimulated with 10 ng/ml murine IFN-γ (a kind gift from G. Adolf, Boehringer Ingelheim, Vienna) or 250 IU/mL of IFN-β (PBL Assay Science; Cat# 12400-1).

#### Chromatin immunoprecipitation (ChIP)

1.5 x 10^7^ bone marrow derived macrophages were seeded on a 15 cm dish on day 7 of differentiation. The next day cells were stimulated for 1.5h either with 10 ng/ml murine IFN-γ (a kind gift from G. Adolf, Boehringer Ingelheim, Vienna) or 250 IU/mL of IFN-β (PBL Assay Science; Cat# 12400-1). Cells were crosslinked for 10 minutes at room temperature in 1 % formaldehyde PBS (Thermo Fisher, Cat# 28906). Cells were quenched with 0.125 M glycine for 10 min at RT. Cells were harvested and washed twice with ice cold PBS. Cells were centrifuged for 5 min at 1350 g at 4°C. Pellets were snap frozen in liquid nitrogen and stored at 80°C overnight. Frozen pellets were thawed on ice for 60 minutes. Pellets were resuspended in 5 mL LB1 (50 mM Hepes, 140 mM NaCl, 1 mM EDTA, 10 % glycerol, 0.5 % NP40, 0.25 % TritonX-100) by pipetting and rotated at 4°C for 10 min. Samples were centrifuged for 5 minutes at 1350 x g at 4°C. Pellets were resuspended in 5 mL LB2 (10 mM Tris, 200 mM NaCl, 1 mM EDTA, 0.5 mM EGTA) by pipetting and rotated at 4°C for 10 min. Samples were centrifuged for 5 minutes at 1350 x g at 4°C. Pellets were resuspended in 3 mL LB3 (10 mM Tris, 100 mM NaCl, 1 mM EDTA, 0.5 mM EGTA, 0.1 % deoxycholate, 0.5 % N-lauroylsarcosine). Samples were split into 2 x 1.5 mL in 15 mL polypropylene tubes suitable for the Bioruptor® Pico (Diagenode). BioRuptor Sonicator settings: power = high, “on” interval = 30 seconds, “off” interval = 45 seconds, 10 cycles. Sonicated samples were centrifuged for 10 minutes at 16000 x g at 4°C to pellet cellular debris. Chromatin concentration was measured by NanoDrop and 25 μg of chromatin were used for each IP. 300 μl 10 % Triton X-100 were added to each 3 mL sonicated lysate. 25 μg of chromatin were stored at 4°C which served later on as an input control. Antibody of interest was added to sonicated chromatin aliquot and mixed (anti-H3K27ac Cell Signaling, Cat# 8173, 5μl; anti-H3K27me3 Active Motif, Cat# 39155, 5ug; IRF9 6F1-H5 supernatant, 150 μl as previously described (Platanitis et al., 2019). All samples were filled up to 1ml with dilution buffer (16.5 mM Tris pH 8, 165 mM NaCl, 1.2 mM EDTA, 1 % Triton X-100, 0.1 % SDS, 0.1 mM PMSF, and complete EDTA-free protease inhibitor cocktail (Sigma Aldrich). Samples were rotated at 4°C overnight.

50 μl of magnetic beads (Dynabeads protein G, Life technologies, Cat# 10003D) per sample were blocked overnight in dilution buffer containing 1 % BSA at 4°C. The next day 50 μl of the beads were added to each sample and incubated at 4°C while rotating. Afterwards the beads were washed 1x with RIPA buffer (50 mM Tris HCl pH 8, 150 mM NaCl, 1 % NP-40, 0.1 % SDS, 0.5 % sodium deoxycholate, 1 mM DTT), 2x high salt buffer (50 Mm Tris pH 8, 500 mM NaCl, 0.1 % SDS, 1 % NP-40), 2x LiCl buffer (50 mM Tris pH 8, 250 mM LiCl, 0.5 % sodium deoxycholate, 1 % NP-40) and TE buffer (10 mM Tris pH 8, 1 mM EDTA) for 10 minutes at 4°C. The samples were eluted in freshly prepared elution buffer (2 % SDS, 100 mM NaHCO_3_, 10 mM DTT). The crosslink between proteins and DNA was reversed by adding 200 mM NaCl to each sample and incubation at 65°C at 300 rpm for 12 hours. Proteinase K, 40 mM Tris pH 8 and 10 mM EDTA were added to each sample and incubated for 1 hour at 55°C and 850 rpm. Each sample was transferred to a phase lock tube (5Prime), mixed 1:1 with phenol-chloroform-isoamylalcohol (PCI) and centrifuged for 5 minutes at 12000 × g. Supernatant was transferred and mixed with 800 μl 96 % Ethanol, 40 μl 3 M CH_3_COONa pH 5.3 and 1 μl Glycogen and stored for at overnight at -20°C. Samples were centrifuged for 45 minutes at 4°C and 16000 × g. Pellets were washed in ice cold 70 % ethanol and dried at 65°C, before diluting the DNA in H_2_O.

Real-time qPCR were run on the Mastercycler (Eppendorf). Primers for ChIP PCR are listed in Table S8.

#### ATAC-seq

3 x 10^6^ BMDM derived from female mice, were seeded in 6-cm non-treated tissue culture plates on day 7 of differentiation. The next day, cells were stimulated for 2h either with 10 ng/ml murine IFN-γ (a kind gift from G. Adolf, Boehringer Ingelheim, Vienna) or 250 IU/mL of IFN-β (PBL Assay Science; Cat# 12400-1).

Cells were gently washed with and scraped in PBS. A viability of >90% was determined with the cell viability analyzer and cell counter NucleoCounter® NC-3000™.

Cells were washed twice in 1 mL ice-cold PBS, by centrifuging cells at 1200 g for 5 minutes. Cells were resuspended in 1 mL cold nuclei isolation buffer (0.32 M sucrose; 3 mM CaCl2; 2 mM Mg Acetate; 0.1 mM EDTA; 10 mM Tris.HCl pH 8.0; 0.6% NP-40; 1mM DTT fresh) and placed on ice for 5 minutes. Cells were centrifuged at 700g for 5 min at 4°C. Supernatant was removed, 500 μL cold nuclei isolation buffer was added and samples were kept on ice for 3 min to further increase the number of nuclei isolated. Cells were centrifuged at 700g for 5 min at 4°C.

Supernatants were removed and pelleted nuclei were gently resuspended in 200 μL cold Nuclei Resuspension Buffer (NRB) (50 mM Tris.HCl pH 8.3; 40% Glycerol; 5 mM MgCl2; 0.1 mM EDTA). 50.000 nuclei (5μl) were resuspended in 7. 5μl H_2_O, 12.5 μl TD buffer and 5 μl Tn5 transposase from Illumina (Cat#15027865).

Nuclei were incubated for 30 min at 37°C, while mixing on a plate shaker (600 rpm).

DNA was purified with Qiagen MinElute columns (Cat# 28006) according to the manufacturer's instructions and eluted in 13μl of water. 12.5 μl purified DNA from the previous step were mixed with 5 μl 2.5 μM I7 index primer, 5 μl 2.5 uM I5 index primer (dual indexing), 2.5 μl Evagreen and 25 μl, 2x Q5 PCR Master Mix, (NEB Cat# M0492L). An end point PCR was run as follows: 5 min 72 ºC, 1 min 98 ºC, 10sec 98 ºC (5-7 cycles) 30sec 65 ºC (5-7 cycles), 60sec 72 ºC (NEB Cat# E7645S).

The reaction was purified by adding 15 μl of SPRI beads prepared by the NGS facility (MBSpure beads) and incubated for 5 min at RT. The supernatant was transferred to a fresh well. 50 μl of MBSpure beads were added, mixed welland incubated for 5 min at RT, magnetized, and the supernatant was removed. The beads were washed twice by adding 150 μl of 80 % EtOH. Beads were dried for 30 sec and DNA was eluted in 20 μl of water. The quality of the libraries was checked on a bioanalyzer to further determine the size distribution. Libraries were sequenced on a NovaSeq6000 S2 PE50.

#### Hi-C

Chromatin interaction libraries were generated using the Arima Genomics (https://arimagenomics.com/) Arima-Hi-C kit (P/N Cat# A510008).

2 x 10^6^ BMDM derived from female mice, were seeded in 6-cm non-treated tissue culture plates on day 7 of differentiation. The next day, cells were stimulated for 2h either with 10 ng/ml murine IFN-γ or 250 IU/mL of IFN-β (PBL Assay Science; Cat# 12400-1). Cells were gently washed with and scraped in PBS. A viability of >90% was determined with the cell viability analyzer and cell counter NucleoCounter® NC-3000™.

Cells were centrifuged at 500 g for 5 min and resuspended in 4.375 mL of 1xPBS at room temperature. 625 μl 16 % formaldehyde were added, mixed and incubated at RT for 10 min. 460 μl Stop Solution 1 was added, mixed well by inverting 10 times and incubated at RT for 5 min. Cells were placed on ice for 15 min and then pelleted by centrifugation (500 g, 5 min, 4°C). Cells were resuspended in 2ml PBS and 1ml (1x10^6^ cells) were pelleted again (500 g, 5 min, 4°C). The pellets were snap frozen in liquid nitrogen. Pellets were resuspended in 20 μl Lysis buffer and incubated at 4°C for 15 min. 24 μl conditioning solution was added, gently mixed and incubated at 62°C for 10 min. 20 μl of Stop solution 2 were added, mixed and incubated for 15 min at 37°C. A mix of 7 μl Buffer A, 1 μl enzyme A1 and 4 μl of enzyme A2 were added to the sample and incubated as follows: 37°C for 60 min, 65°C 20 min, 25°C 10 min. 12 μl of Buffer B and 4 μl of enzyme B were added to the mix and incubated for 45 min at RT. Further 70 μl Buffer C and 12 μl enzyme C were added, mixed gently and incubated for 15 minutes at RT. 10,5 μl Buffer D and 25 μl enzyme D were mixed. 20 μl Buffer E were added and the whole mix was added to the sample and incubated as follows: 55°C for 30 min, 68°C for 90 min, 4°C hold. 100 μl of MBS pure beads were added and incubated at RT for 5 min. The supernatants were removed from the magnetized solution. 200 μl 80 % EtOH were added while the plate was still on the magnet. EtOH was removed, beads were resuspended in 50 μl elution buffer, and incubated at RT for 5 min. The eluate was transferred to a new tube and the DNA concentration was determined by Qubit. 5 μg DNA of each sample were fragmented by using the Covaris S2 and the following program: Intensity - 5, Duty Cycle - 10%, Cycles per burst – 200, Treatment Time - 50 sec, Temperature – 7 ˚C. 50 μl of MBS pure beads were added to the 100 μl of sheared solution and incubated for 5 min at RT. Supernatants from magnetized solutions were transferred to a fresh well. 50 μl of beads were added and incubated for 5 min at RT. Supernatant was removed from the magnetized solution and 200 μl 80 % EtOH were added, while the plate was still on the magnet. EtOH was removed and beads were resuspended in 100 μl elution buffer. The DNA amount was quantified by Qubit, followed by the Biotin enrichment protocol and library preparation. 300ng of DNA were used for the library preparation using the Ultra II kit (NEB, Cat# E7103S) for adaptation. 100 μl of enrichment beads were added and incubated at RT for 15 min. Supernatant was discarded, beads were resuspended in 180 μl wash buffer and incubated at 55°C for 2 min. The washing step was performed again. Beads were resuspended in 100 μl elution buffer and the supernatant was again removed. Beads were resuspended in 50 μl elution buffer, 7 μl End prep buffer and 3 μl end prep enzyme were added and the mix was incubated 30 min at 20 ˚C and 30 min at 65 ˚C. 30 μl Ligation master mix, 2.5 μl Adapter and 1 μl ligation enhancer were added and incubated at RT for 15 min. The beads were magnetized and the supernatant was discarded. Beads were washed twice in 180 μl Wash buffer, while incubated at 55°C for 2 min. Beads were resuspended in 100 μl elution buffer at RT. Supernatants were removed and beads were resuspended in 12 μl of elution buffer. 3 μl of USER enzyme, 10 μl i5/i7 index primer mix (2.5 μM), 2.5 μl Evagreen and 25 μl Q5 PCR mix from NEB ultra II Kit (Cat# E7103S) were added and the PCR was run as follows: 37°C 15 min, 98°C 30 sec, 98°C 10 sec, 65°C 75 sec (6 cycles endpoint PCR). Libraries were purified with 1x MBS pure beads. Libraries were sequenced on a NovaSeq6000 S1 PE50.

#### Hi-C data processing

Hi-C data were processed using the Hi-C pipeline from the nf-core framework (nf-core/Hi-C v1.1.0; https://zenodo.org/record/2669513#.YA8UvWRKjZk; (Ewels et al., 2020)). Reads were aligned against the Illumina iGenome *Mus musculus* GRCm38 reference genome. Restriction sites used were ˆGATC, GˆANTC. Ligation sites used were GATCGATC, GANTGATC, GANTANTC, GATCANTC.

#### HiCreppy

Sample similarity for Figure S1A was assessed using hicreppy (v0.0.6) (https://github.com/cmdoret/hicreppy), a reimplementation of HiCRep (https://doi.org/10.1101/gr.220640.117), for Hi-C maps with 10 kb bins and a maximum scanning distance of 1 Mb. The algorithm is computing a stratum-adjusted correlation coefficient, which was used for evaluating reproducibility across replicates and conditions.

#### PCA/compartment analysis

The first three eigenvectors of Hi-C contact maps were computed with *cooltools.eigdecomp* (cooltools, v0.3.0) using bedgraph tracks from published RNA-seq data (Platanitis et al., 2019) to phase and rank them according to their correlation. The eigenvector showing the highest correlation with the RNA-seq data was considered the one representing A and B compartments. Vectors were processed independently for each chromosome. In order to measure the compartmentalization strength, we generated compartmentalization plots (“saddle plot”) with Hi-C maps at 80 kb resolution using cooltools, as described in their manual (https://cooltools.readthedocs.io/en/latest/index.html). Compartmentalization changes at specific regions for Figure 1E were quantified by calculating the difference between the first eigenvectors of treated and untreated conditions (merged replicates) at a resolution of 40 kb. Regions of interest (clusters from Figure 2) were extracted. Size-matched control regions, 1 Mb upstream and downstream of each region were defined. Bins from the first eigenvectors, overlapping these regions were extracted and for each region, the mean differences of the overlapping bins were calculated and plotted. For compartmentalization analysis in specific groups of genes in Figures 4B and 4C, the same analysis was performed by using the respective gene regions as regions of interest. Statistics in Figures 4B and 4C: Wilcoxon rank-sum test for unpaired data.

#### Ratio plots

Ratio plots, comparing interaction strengths between two conditions (Figures 1B and 1D) were generated using Serpentine (Baudry et al., 2020), a flexible 2D binning method improving especially the comparison of poorly covered and noisy regions. It is operating on raw data and is only performing binning where necessary on noisy regions, while at the same time preserving the resolution of highly covered regions.

#### Pattern detection

Pattern detection in the Hi-C contact maps was performed using Chromosight (v1.3.3) (Matthey-Doret et al., 2020). Chromosight is sliding pattern templates over contact maps and, for each position, computing Pearson correlation coefficients between the template and the contact map.

In order to make the correlation scores for called patterns comparable between replicates and conditions, a fusion map was generated containing the contacts of all replicates across conditions with *cooler* (v0.8.10) (Abdennur and Mirny, 2020). A first round of pattern detection was performed on this fusion map with *chromosight detect* on contact maps with bin sizes of 5 kb and 10 kb at a *maximum scanning distance* of 10 Mb. The pattern used was *loops_small.* The positions of detected patterns were passed to *chromosight quantify* for quantification of Pearson correlation scores in individual contact maps for each condition. Therefore, contact maps were subsampled to the number of contacts in the smallest matrix to ensure equal coverage across conditions. For Figures 2A–2F, 3A, 3B, S2 and S3A–S3C detected patterns were filtered for Pearson scores > 0.35.

#### Reproducibility of pattern detection

In order to determine a reproducibility measurement for pattern detection, correlation scores for loops overlapping shown ISG clusters (Figures 2 and S3) (i.e. at least one anchor inside the cluster intervals), with a length < 500 kbp were considered. A Spearman correlation score was calculated for loop score correlation between individual samples/replicates and visualized in a heatmap (Figure S2C).

#### Inter-/Intracluster loop strength ratio calculations

From all called loops, the ones overlapping indicated regions of interest were considered and classified as inter- (one anchor inside regions of interest) and intra- (both anchors inside region of interest) cluster loops. Pearson correlations from *chromo sight* were used as an indicator for loop strength. Plots in Figures 3A–3D show all datapoints, for the heatmap in Figure 3E ratios between the medians of inter- and intracluster loops were calculated.

#### ATAC-seq data processing

ATAC-seq data was processed using the ATAC-seq pipeline from the nf-core framework (nf-core/atacseq v1.2.1, https://zenodo.org/record/3965985#.YA8a8GRKjZk). Reads were aligned against the Illumina iGenome *Mus musculus* GRCm38 reference genome. Downstream analysis of ATAC-seq data was performed using MACS2 narrow-peak calling and differential chromatin accessibility analysis (included in nf-core/atacseq pipeline).

#### ATAC-seq heatmaps

For the generation of summary profile plots and heatmaps, density information (bigwig) for gene regions and surrounding regions (2 kbp upstream of TSS and 1 kbp downstream of TES) was plotted using *deeptools* (v3.4.3) (https://zenodo.org/record/3965985#.YA8XPGRKjZk). For defining the groups of ISG in Figures 5A–5D, the top 200 significantly (p-value ≤ 0.05) upregulated genes (sorted by log2FC) according to RNA-seq analysis were filtered. The second cluster refers to the respective remaining genes in the genome. For the grouping in Figures 5E–5H, genes were filtered according to log2FC as indicated. For the grouping in Figures S4D and S4E, genes were filtered for being significantly induced by one, but not the other stimulus (log2FC ≥ 1, adjusted p-value ≤ 0.05). The *computeMatrix* command was used in the *scale-regions* mode with the option *missingDataAsZero*. Subsequently, *plotHeatmap* was used for the generation of heatmaps and summary profiles.

#### ATAC-seq integration with RNA-seq

For integrating ATAC-seq and RNA-seq we used the respective differential analyses from DESeq2. For ATAC-seq, each called interval (available output from the nf-core pipeline) was annotated to a gene by using the HOMER script *annotatePeaks.pl*. (Heinz et al., 2010) Thereby peaks were annotated to genes and could be compared with the RNA-seq analysis.

#### Interaction strengths between regulatory regions

In order to calculate interaction strengths between regulatory regions, ATAC-seq and Hi-C data was integrated. ATAC-seq peaks (accessible regions), for each treatment overlapping all regions of interest (*Gbp, Cxcl, Ifit, Irf1, Irf8, Mx, Oas, Rnf213* clusters) were included in the analysis. Since ATAC-seq peaks overlapping gene bodies tend to be broad in some cases and therefore not informative for regulatory elements, those were excluded. Interaction strengths between all possible peak pairs annotated to the same cluster were calculated from Hi-C data with the *cooler* software. Additionally, control pairs were calculated as follows. For each peak pair (A-B, with B being located downstream of A), two control pairs were obtained. One between A and a position upstream of A, having the same distance and size as position B. The other one between B and a position downstream of B, having the same distance and size as position A. To compare “real” and “control” interaction strengths, the mean of the two “control” positions for each “real” position was calculated and plotted. Statistical analysis was performed with Wilcoxon rank-sum test for paired data.

#### ChIP-seq data processing

Published ChIP-seq data (Mancino et al., 2015; Platanitis et al., 2019; Wienerroither et al., 2015) were re-analysed using the ChIP-seq pipeline from the nf-core framework (nf-core/chipseq v1.1.0) (https://zenodo.org/record/3529400#.YA8YGmRKjZk). Reads were aligned against the Illumina iGenome *Mus musculus* GRCm38 reference genome.

### Quantification and statistical analysis

Site directed ChIP data represent the mean values with standard deviation (SD). Statistical significance was calculated using two-tailed unpaired t-test. All statistical analysis of ChIP data was performed using GraphPad Prism (Graphpad) software. Asterisks denote statistical significance as follows: ns: p > 0.05, ∗: p ≤ 0.05, ∗∗: p ≤ 0.01, ∗∗∗: p ≤ 0.001. Each dot represents one biological replicate.

## Data Availability

Data: Publicly available raw data processed in this paper are available under accession numbers GEO: GSE115435 (IRF9 ChIP-seq), ArrayExpress: E-MTAB-2972 (Pol II ChIP-seq), GEO: GSE56123 (H3K27Ac). Raw data generated for this publication in HiC and ATAC-seq experiments are available under SRA BioProject: PRJNA694816. Code: The paper does not report original code. All software and pipelines used in our study are listed and referenced in the key resources table.

## Supplemental information

Document S1. Figures S1–S5 and Table S8

Table S1. Analysis of compartment strength (PC1 differences) in control and IFN-treated macrophages, related to figures 1E, 4B, 4C. Values underlie the calculation of PC1 differences

Table S2. Determination of intra- and extracluster loop strength, related to figures 3A-D. Values underlie the loop scores shown in the figures

Table S3. Differential analysis of IFN-I- and IFN-γ-induced transcriptome changes as determined by RNA-seq, related to figure 4A. Values underlying were used to generate the volcano plot

Table S4. Analysis of chromatin interaction strength between regulatory or control regions, related to figure 5I. Values underlying the interaction strength scores shown in the figure

Table S5. Integrated Analysis of IRF9-dependent changes in chromatin accessibility (ATAC-seq) and mRNA expression (RNA-seq) in resting macrophages, related to figure 6A. Values were used to generate the volcano plot

Table S6. Integrated Analysis of IFN-I-induced changes in chromatin accessibility (ATAC-seq) and mRNA expression (RNA-seq) in wt and IRF9-deficient macrophages, related to figures 6B, D. Values were used to generate the volcano plot

Table S7. Integrated Analysis of IFN-γ-induced changes in chromatin accessibility (ATAC-seq) and mRNA expression (RNA-seq) in wt and IRF9-deficient macrophages, related to figures 6C, E. Values were used to generate the volcano plot
