# Supplementary material for: Interferons reshape the 3D conformation and accessibility of macrophage chromatin
Source: iScience. 2022 Feb 1;25(3):103840. doi: 10.1016/j.isci.2022.103840 (PMC8857492; doi:10.1016/j.isci.2022.103840)
Supplement: Document S1. Figures S1–S5 and Table S8 [file mmc1.pdf]

iScience, Volume *25*

Supplemental information

Interferons reshape the 3D conformation and accessibility of macro-phage chromatin

Ekaterini Platanitis, Stephan Gruener, Aarathy Ravi Sundar Jose Geetha, Laura Boccuni, Alexander Vogt, Maria Novatchkova, Andreas Sommer, Iros Barozzi, Mathias Müller, and Thomas Decker

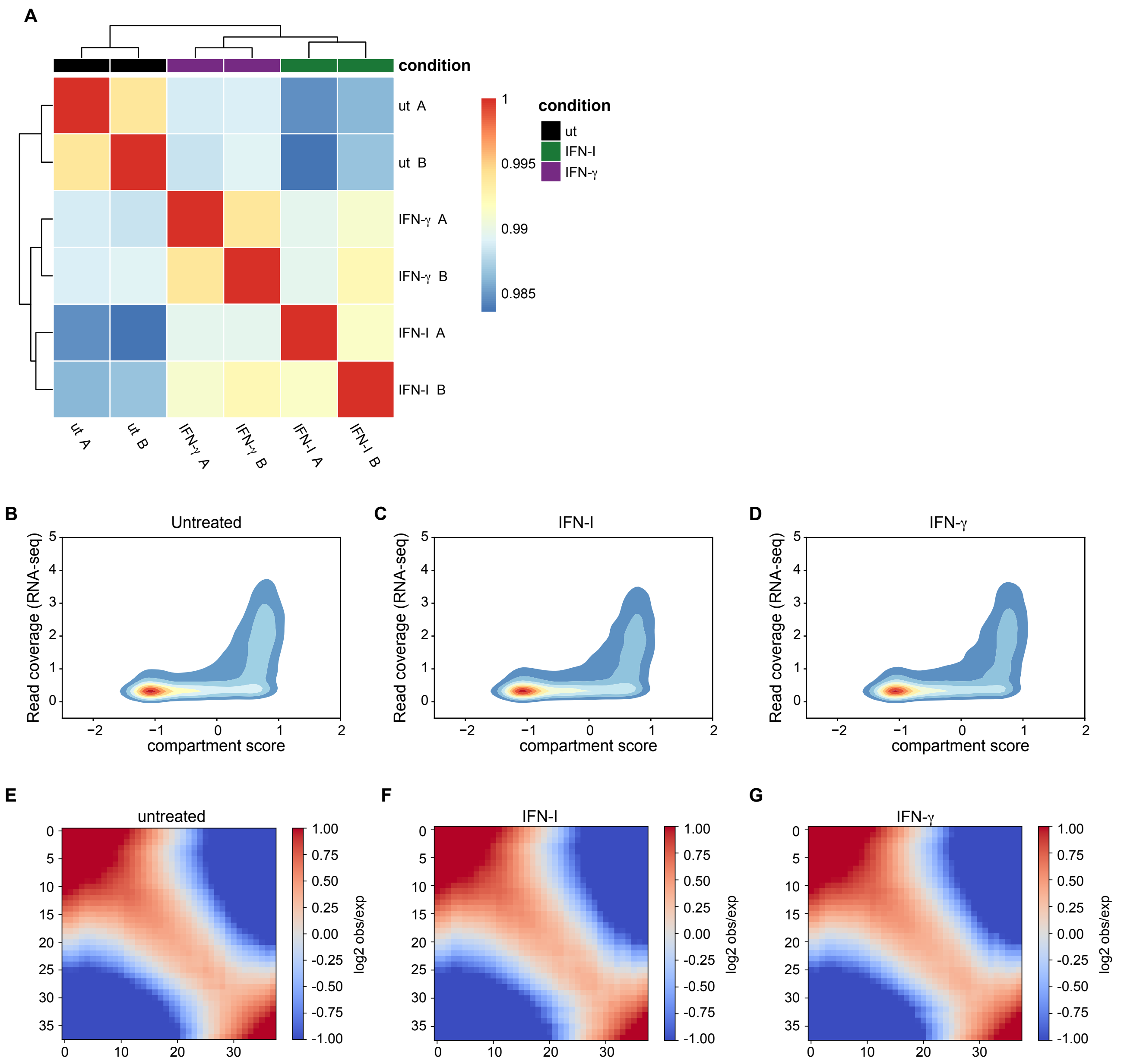

Figure S1. Quality control of HiC data. Related to figure 1. A Heatmap of sample similarity, as calculated with HiCRep (max. scanning distance 10Mbp, 10kbp bins). B-D Density plots of RNA-seq read coverage versus compartment score for each genomic bin (40kbp) of B untreated, C IFN-I treated (2h), D IFN-γ treated (2h) primary murine bone marrow-derived macrophages (BMDM). E-G Compartmentalization saddle plots showing the enrichment of interactions as a function of the compartment vector in E untreated, F IFN-I (2h) and G IFN-γ (2h) treated primary murine bone marrow-derived macrophages (kbp bins).

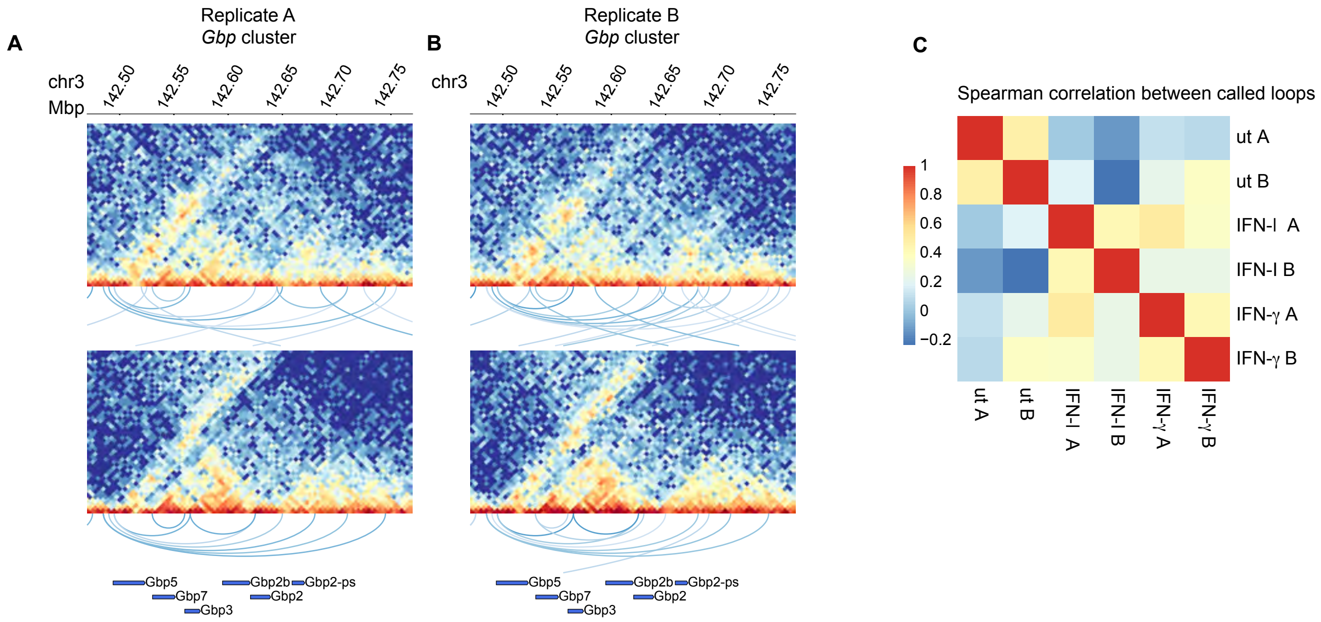

Figure S2. Correlation between HiC data replicates and correlation between loops called in individual samples. Related to figure 2. A, B Hi-C contact maps of untreated and IFN-I treated BMDM. Visualization of pattern detection with chromosight (loops between two loci are indicated as arcs). Color coding of arcs corresponds to Pearson correlation scores. Loops with a score > 0.35 are shown (maximum size/ylim = 500 kbp). Only ISG of the corresponding gene clusters are visualized. Single replicates are plotted next to each other. Only ISG of the corresponding gene clusters are visualized. C Spearman correlation of loop scores at ISG loci shown in Figures 2, 3, S3 (Gbp, Cxcl, Ifit, Irf1, Irf8, Oas, Mx, Rnf213) between individual samples.

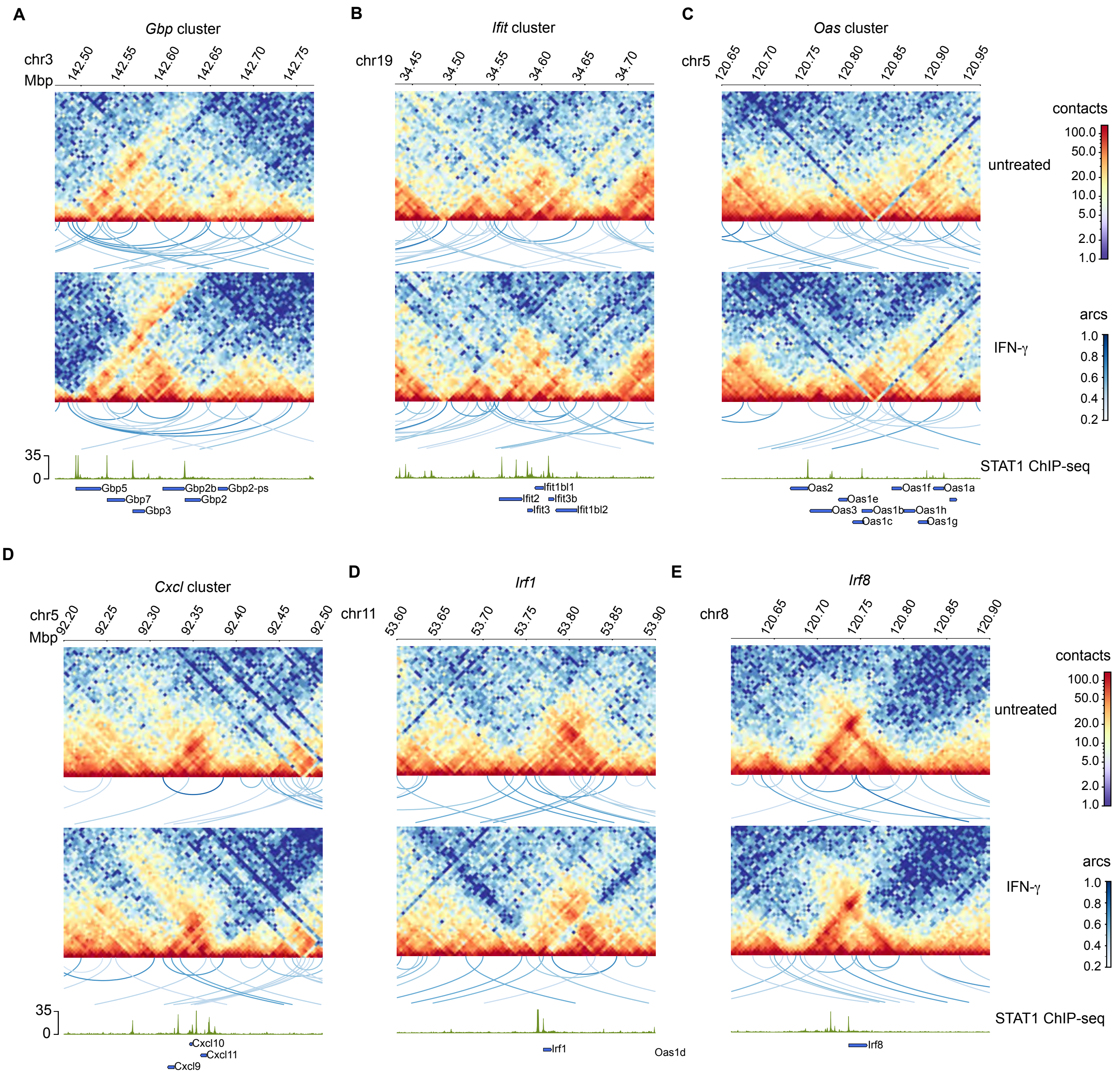

Figure S3. Effect of IFN-γ on the loop structure of ISG loci. Related to figure 2. A-F Hi-C contact maps (merge of 2 replicates per condition, log1p scale) of untreated and IFN-γ (2h) treated BMDM. Visualization of pattern detection with chromosight (loops between two loci are indicated as arcs). Color coding of arcs corresponds to Pearson correlation scores. Loops with a score > 0.35 are shown (maximum size/ylim = 500 kbp). Lower panel, STAT1 ChIP-seq tracks (1.5h IFN-γ treatment) and gene annotations are shown to visualize binding sites for both ISGF3 and STAT1 homodimers. Only ISG of the corresponding gene clusters are visualized.

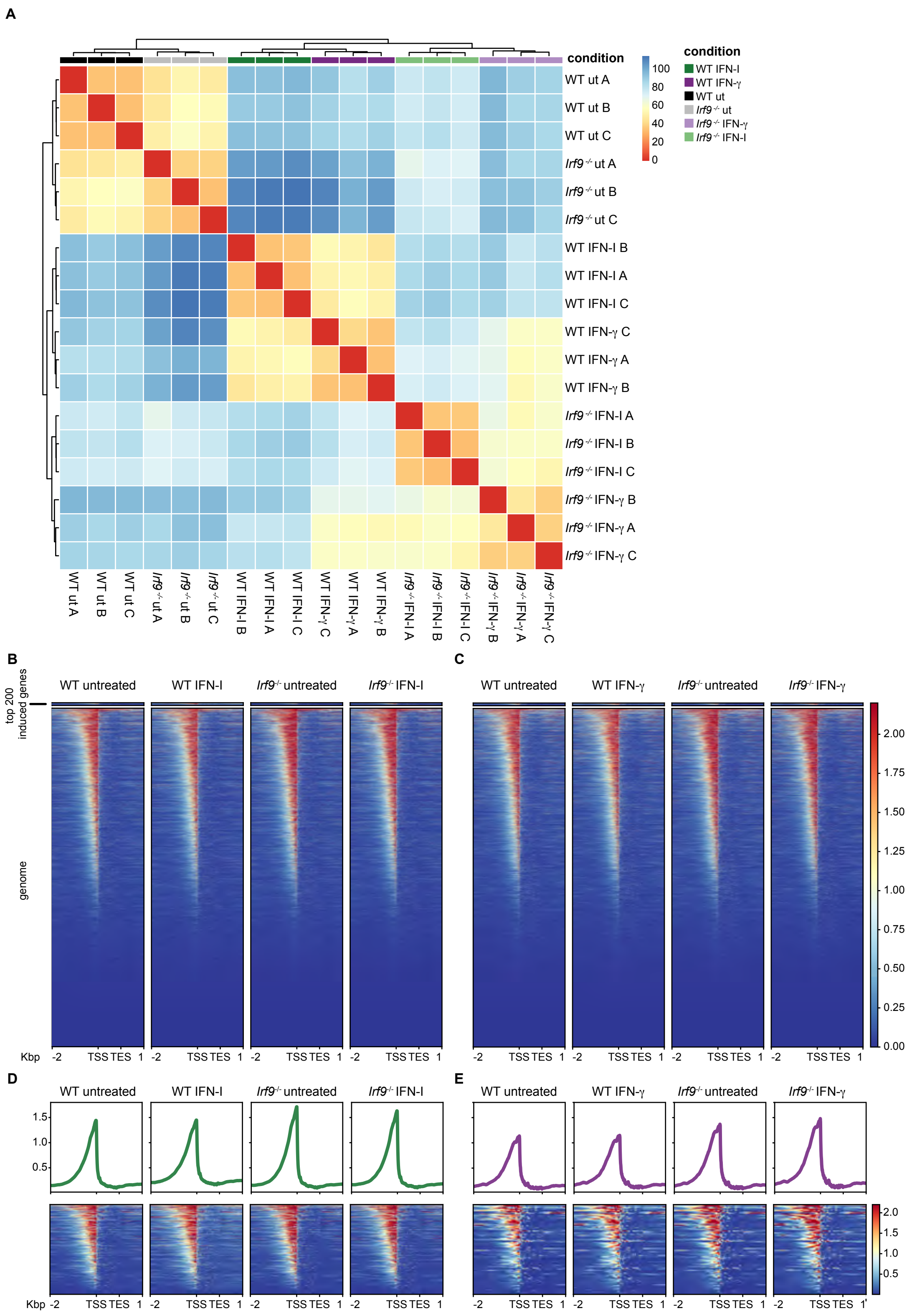

Figure S4. Similarity of ATAC-seq samples and effect of IFN and IRF9 deficiency on chromatin opening. Related to figures 4, 5 and 6. A Heatmap of sample similarity, generated from clustering by Euclidean distances between DESeq2 rlog values for each sample. B, C Heatmaps of chromatin accessibility in regions of the top 200 genes (log2FC) upregulated after IFN-I (2h) and IFN-γ (2h) treatment respectively according to RNA-seq and the remaining genome. Heatmaps are shown for the regions of the genes, including 2 kbp upstream of the TSS and 1kbp downstream of the TES as indicated below the plots. Data are derived from three biological replicates. D, E ATAC-seq intensities at TSS of genes specifically induced by IFN-I (D) or IFN-γ (E) according to RNA-seq. Top panels: Summary profile indicating the average ATAC-seq signal over all regions. Lower panels: Heatmaps of chromatin accessibility.

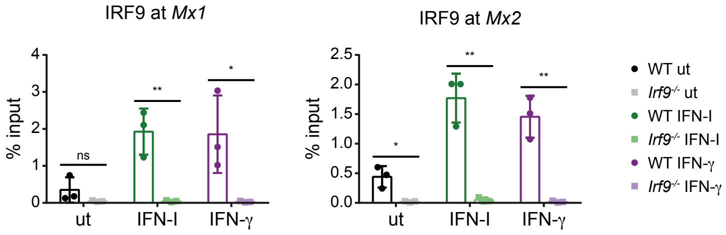

Figure S5. Control ChIP-qPCR showing association of IRF9 with the Mx1 and Mx2 promoters. Related to Figure 7. Site directed ChIP at the Mx1, Mx2 gene promoters was performed with anti-IRF9 mAb using bone marrow-derived macrophages isolated from wild-type and Irf9-/- BMDM, treated for 2h either with IFN-I or IFN-γ.

ChIP PCR Primers		
Gene name	Forward sequence 5'-3'	Reverse sequence 5'-3'
Oas1a	ATACAGAGCCCCACTCACAACT	TCTGGGTCTGTCGCATCG
Oas2	ATCCACGGCCTGGTCTATCT	TGTCTGAAGCAGATTGCGGT
Irf1	TCCCGCTAAGTGTTTAGATTTC	TTCGGTTCGGCTTAGACTG
Mx1	GCTCAGGCTTTTTCACGGTTTCA	GGCTTGAAGAGAGAGTGTGCAG
Mx2	CTTCTGCCCAGAATCAGGC	AGTTTCACTTTCATTTCTCTGGTT
untr6	TCAGGCATGAACCACCATAC	AACATCCACACGTCCAGTGA

Table S8: Primers used for ChIP-Q-PCR. Related to STAR methods.
